# Vaginal metastasis in solid tumours: our four cases and review of the literature

**DOI:** 10.1186/s43046-021-00058-4

**Published:** 2021-02-04

**Authors:** Mustafa Korkmaz, Melek Karakurt Eryılmaz, Ülkü Kerimoğlu, Mustafa Karaağaç, Aykut Demirkıran, Emine Türen Demir, Mehmet Artaç

**Affiliations:** 1 Department of Medical Oncology, Necmettin Erbakan University School of Medicine, Akyokuş, 42080 Konya, Turkey; 2 Department of Radiology, Necmettin Erbakan University School of Medicine, Konya, Turkey; 3 Department of Department of Obstetrics and Gynecology, Necmettin Erbakan University School of Medicine, Konya, Turkey

**Keywords:** Vaginal metastasis, Gynaecological malignancies, Colorectal cancers

## Abstract

**Background:**

Vaginal metastasis should be kept in mind when evaluating the staging tests of all cancers, especially endometrial cancer.

**Case presentation:**

We present four patients with vaginal recurrence who recently applied to our clinic. Three cases were of endometrial cancer and one case of rectal cancer. All patients presented with vaginal bleeding.

**Conclusion:**

Standard treatment for vaginal metastasis has not yet been established. Therapeutic options for vaginal metastasis—separately or in combination—are surgical resection, radiotherapy, and chemotherapy.

## Background

Endometrium carcinoma (EC) is one of the most common gynaecological malignancies and the fourth most common malignancy of women in the USA. Although surgery is the mainstay of treatment, the recurrence of EC after surgical treatment remains a major problem in high-risk patients. Despite adjuvant chemotherapy (CT) in high-risk patients, local or pelvic recurrences occur frequently in the vagina, pelvic nodes, and to a lesser extent in the cervix, adnexa, and peritoneum [1].

Colorectal cancers (CRC) are the second most common type of cancer worldwide that can develop from any part of the large intestine, especially the rectum. Distant metastases occur in 20–30% of patients with CRC, and the most common sites of distant metastasis are the liver and lung, but the vagina has been rarely reported as a site of metastasis [2].

We presented four cases of vaginal metastasis, three of which originated from EC and one from rectal cancer, and we reviewed the literature on vaginal metastasis.

## Case presentation

### Endometrial cancer with vaginal metastasis at diagnosis

#### Case 1

**Fig. 1:**
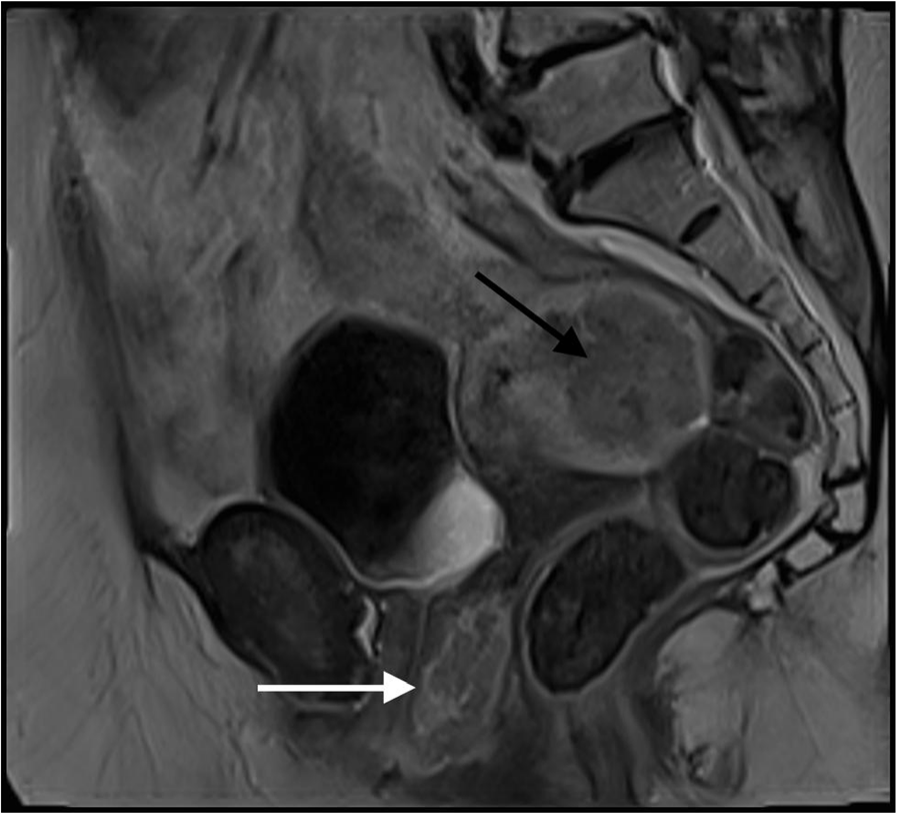
Sagittal T1-weighted image obtained after administration of contrast material demonstrates endometrial mass (black arrow) with heterogeneously enhanced vaginal metastasis (white arrow)

A 66-year-old woman applied with vaginal spotting in October 2019. On vaginal examination, a normal and healthy cervix and vagina were detected. Vaginal smear was not compatible with malignancy. Pelvic magnetic resonance imaging (MRI) showed a 4.5 × 4.5 centimetre (cm) intrauterine mass and a second 4 cm mass located in the distal 1/3 of the vagina (Fig. 1). Squamous cell carcinoma (SCC) was detected in fractional endometrial curettage. However, no malignancy was reported in the pathological evaluation of the first biopsy of the vaginal mass. Then, a total abdominal hysterectomy was performed with bilateral salpingo-oophorectomy and bilateral pelvic-paraaortic lymph node dissection (TAH+BSO+BPPLND). Histopathological examination revealed grade 2 endometrioid type EC with squamous differentiated. The intrauterine tumour occupied more than half of the myometrium. There was no involvement in the lymph nodes, cervix, paracervical, or parametric region. The surgical margins were intact and there was no lymphovascular invasion (LVI). Due to the ongoing clinical suspicion of malignancy related to the vaginal mass, a second biopsy was performed 2 months after the surgery, and malignant epithelial neoplasm was reported at the end of the pathological examination. In the multidisciplinary tumour board, it was discussed whether the patient had a vaginal metastasis from EC or primary vaginal cancer. Thereafter, surgery was performed for the vaginal mass. Histopathological examination of the vaginal mass was consistent with differential squamous EC metastasis. All pathology specimens were consulted to a second centre and the same findings were confirmed. Thus, the patient was defined as vaginal metastatic EC at the time of diagnosis. Adjuvant chemotherapy including 6 cycles of carboplatin plus paclitaxel was applied followed by radiotherapy (RT) consisting of 180 centigray (cGy) per day for a total dose of 4500 cGy in 25 fractions. And then, brachytherapy was performed.

### Recurrent endometrial cancer with isolated vaginal metastasis

#### Case 2

**Fig. 2:**
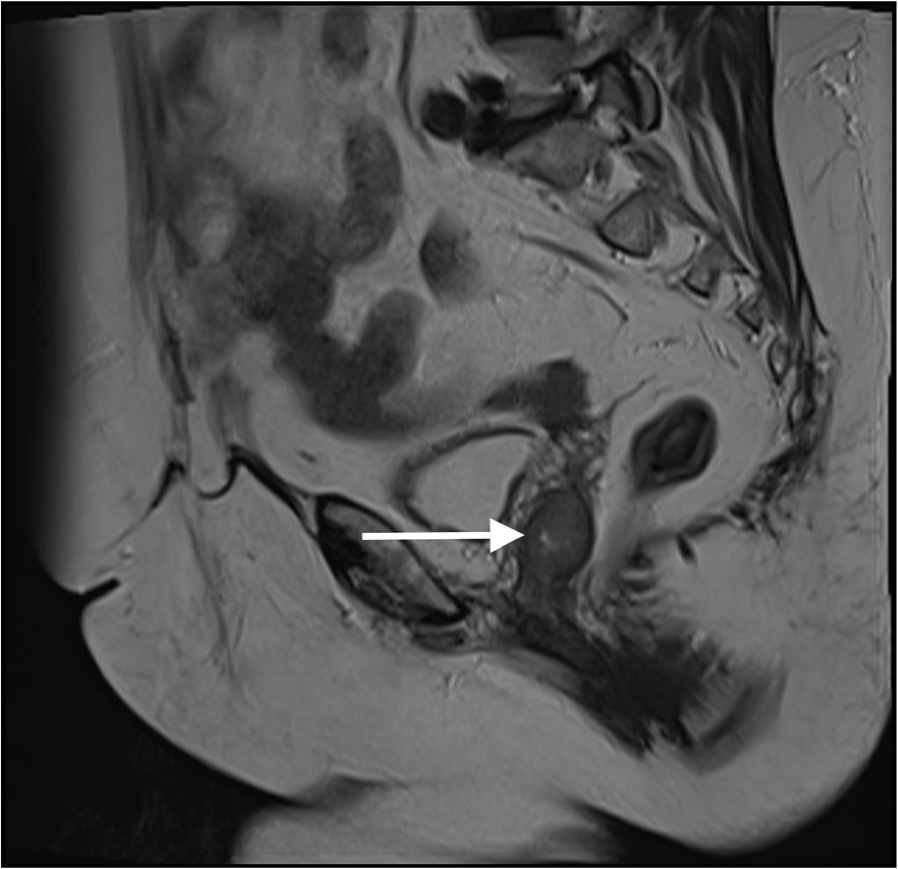
Sagittal T2-weighted image demonstrates lesion (arrow) with intermediate T2 signal intensity filling vaginal lumen

A 66-year-old woman presented with abdominal pain and vaginal bleeding in May 2018. A well-differentiated endometrioid type EC was revealed by endometrial biopsy. TAH+BSO+BPPLND was applied and a grade 1 endometrioid type EC was reported in histopathological examination. EC was staged as T1aN0M0-Stage 1A, FIGO grade 1 without LVI. No adjuvant therapy was applied after the surgery. Two years later, the patient was admitted with bloody vaginal discharge and vaginal bleeding. A 23 × 20 mm nodular mass was found in the left wall of the vaginal cuff on pelvic MRI (Fig. 2). There were no other metastatic lesions on the thorax and abdominal computed tomography (CT). A biopsy was performed from the vaginal mass and EC metastasis was reported after the pathological examination. Surgery was not planned, and RT consisting of 180 centigray (cGy) per day for a total dose of 5040 cGy in 28 fractions was performed.

#### Case 3

**Fig. 3:**
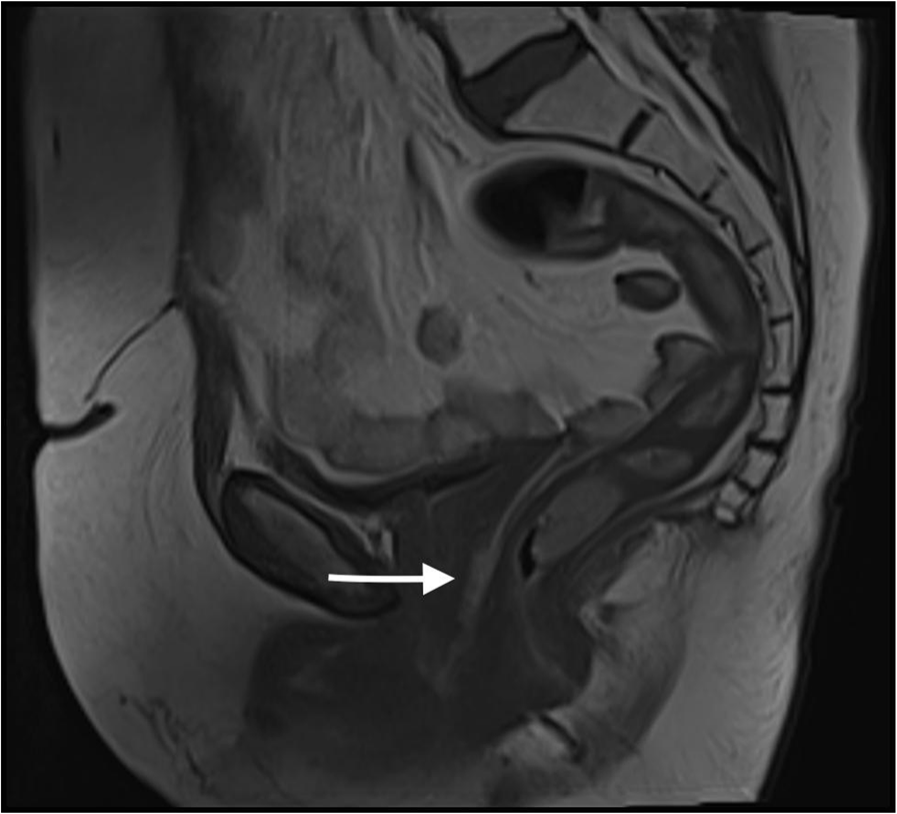
Sagittal T1-weighted image obtained after administration of contrast material demonstrates longitudinal thickening and enhancement of vaginal walls distally (arrow)

A 69-year-old woman with no significant medical history and no previous hormone replacement therapy presented with postmenopausal bleeding in September 2019. A grade 1 endometrioid type EC was detected with an endometrial biopsy. Therefore, TAH+BSO+BPPLND was performed and a grade 2 endometrioid type EC was showed via histopathological examination. And, EC was staged as T1bN0M0-Stage 1B, FIGO grade 2 without LVI. Thereafter, adjuvant brachytherapy was performed. Four months after the completion of brachytherapy, the patient was presented with vaginal bleeding. There was no lesion on vaginal examination, and pelvic MRI showed only 5–6 mm of thickness on the posterior wall of the vagina (Fig. 3). A vaginal biopsy was done and endometrioid type EC was detected. Thorax and abdominal CT showed no other metastatic lesions. Therefore, RT consisting of 180 centigray (cGy) per day for a total dose of 4500 cGy in 25 fractions was planned and performed.

### Recurrent rectal cancer with vaginal metastasis

#### Case 4

**Fig. 4:**
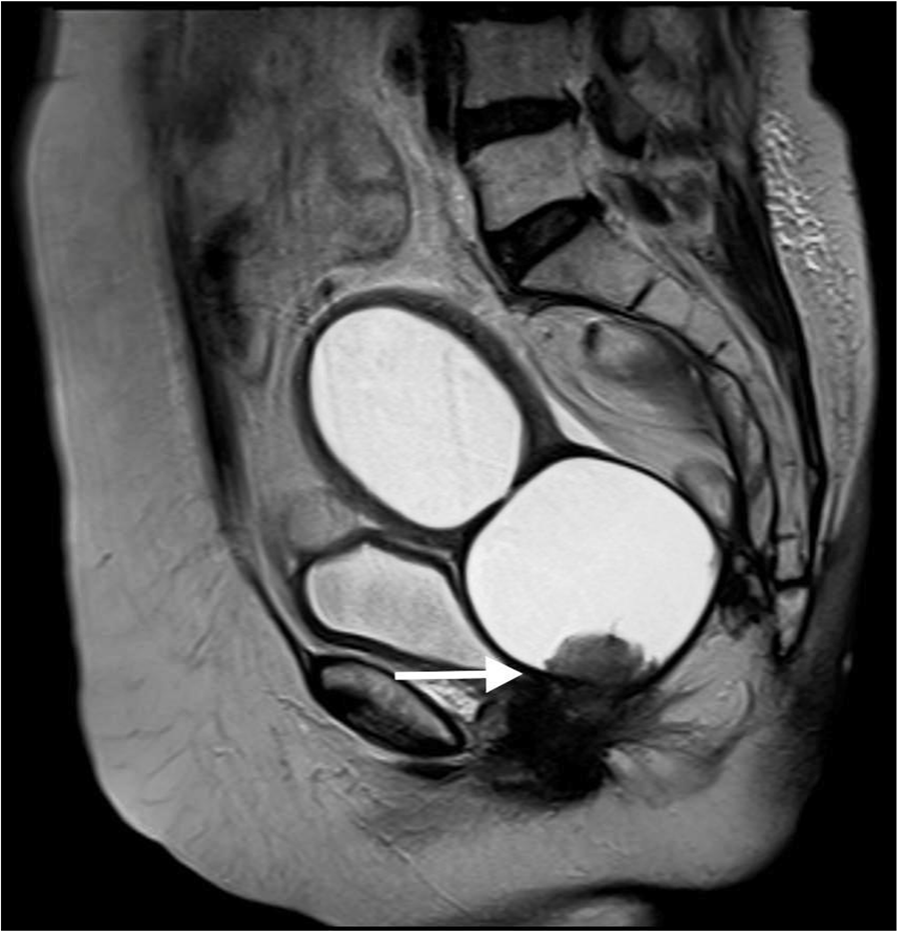
Sagittal T2-weighted image demonstrates increased T2 signal intensity representing water, filling endometrial and vaginal lumen secondary to obstruction of metastatic lesion (arrow) at the vaginal lumen distally

A 66-year-old woman who presented with bloody stools in June 2017 was diagnosed with rectal cancer after the colonoscopic biopsy. There was no distant metastasis on the thorax and abdominal CT, and laparoscopic low anterior resection was performed after neoadjuvant chemoradiotherapy. And, the pathological examination showed moderately differentiated mucinous adenocarcinoma of the rectum that invaded the proper muscle. The size of the tumour was 2.2 × 2 cm, there was no lymph node metastasis within the 14 resected lymph nodes, and rectal cancer was staged as ypT2N0M0. Although the perineural invasion was observed, LVI was not detected. Then, twelve cycles of adjuvant mFOLFOX-6 chemotherapy regimen containing folinic acid, 5-Fluorouracil, and oxaliplatin were performed biweekly. During the follow-up period in January 2019, a 3.5 × 2 cm mass was detected in the anterior part of the vagina on pelvic MRI (Fig. 4). Then, a vaginal biopsy was done, and colon adenocarcinoma metastasis was reported. Although the perineural invasion was observed, LVI was not detected. Then, twelve cycles of adjuvant mFOLFOX-6 chemotherapy regimen containing folinic acid, 5-Fluorouracil, and oxaliplatin were performed biweekly. During the follow-up period in January 2019, a 3.5 × 2 cm mass was detected in the anterior part of the vagina on pelvic MRI (Fig. 4). Then, a vaginal biopsy was done, and colon adenocarcinoma metastasis was reported. Thorax and abdominal CT showed no other metastatic lesions. The case was discussed at the multidisciplinary tumour board, the decision was made for surgery, and eventually, the vaginal mass was resected in March 2019. In the final pathological assessment, colonic adenocarcinoma metastasis was reported as expected. Then, twelve cycles of adjuvant mFOLFOX-6 chemotherapy regimen were re-performed biweekly.

## Discussion

### Methods

References from relevant articles for this review were identified by a search of PubMed using the term vaginal metastasis. All patients with metastasis to the vagina were classified according to the primary tumour areas, and patient characteristics were discussed.

#### Vaginal recurrence of colorectal cancer

Vaginal metastasis from CRC is very rare, and only 58 cases have been reported in the literature. Data were available for only 23 of these patients, while data for the remaining 35 patients were not available. The first clinical sign of vaginal metastasis in most patients was vaginal bleeding. Only 20 patients’ information on stages at the time of diagnosis was reported, and 5% (1/20) had stage I, 30% (6/20) had stage II, 50% (10/20) had stage III, and 15% (3/20) had stage IV disease. Forty-four percent of patients (10/23) whose data were available had metastases in other areas in addition to vaginal metastases. RT, surgery, and chemotherapy treatments were applied in various combinations to these patients with multiple metastases. And, 56% of patients (13/23) had isolated vaginal metastasis. The mean time from the first diagnosis of CRC to the detection of isolated vaginal metastasis in these patients was 15.2 months (range 3–24). Transvaginal wide excision was performed in 11 of 13 patients with the isolated vaginal metastases, and external beam RT (EBRT) was applied to the remaining 2 patients. Of the 11 patients who underwent the transvaginal wide excision, 3 received postoperative chemotherapy, 5 received postoperative RT, and the remaining 3 were followed without additional therapy [3].

#### Vaginal recurrence of renal cell cancer

Metastasis to the vagina from renal cell cancer (RCC) is extremely rare. The first case of RCC-originated vaginal metastasis described in the literature was reported by Penham in 1906, and less than 100 such cases have been reported in the medical literature to date. A firm solitary lesion was found in the lower third of the anterior vaginal wall in most of these patients. While vaginal metastasis in most of the cases was reported as a metachronous metastatic disease in a long-term period after radical nephrectomy, synchronous vaginal metastasis was described in only four patients [4]. The median age at diagnosis in the reported cases was 57 years (range 14–88). Clinical presentation in 65% of cases was due to vaginal leaking, bleeding, or mass effect. Vaginal metastases were usually solitary, and lesion size varied between 0.5 and 8 cm. The median survival was 19 months (range 1–96). Local excision and RT are the recommended treatment modalities for solitary vaginal metastasis in patients previously treated with radical nephrectomy [5].

#### Vaginal recurrence of gastric cancer

In the current literature, there is only one case with isolated vaginal metastasis of gastric cancer. The patient was operated with negative surgical margins for gastric cancer. Metastases were detected in the patient’s vagina and bladder wall in the 5th month after surgery, and chemotherapy was applied [6].

#### Vaginal recurrence of urothelial cancer

Metastasis of urothelial cancer to the vagina is extremely rare, and only 15 cases have been reported worldwide. While muscle invasion was reported in the surgical material of primary urothelial carcinoma in four of these cases, it was not reported in the remaining 11 patients. Synchronous metastasis developed in 2 patients and metachronous metastasis in 13 patients. The median time from urothelial cancer diagnosis to vaginal metastasis was 4.3 years (range 0–14). Endoscopic resection was performed in 11 patients, and total hysterovaginectomy in 2 patients. The prognosis was good, and relapse was rare in these cases [7].

#### Vaginal recurrence of bladder cancer

Vaginal cuff recurrence occurred in 34 of 469 women (7.3%) treated with radical cystectomy for bladder cancer. The 5-year overall mortality-free survival rate was 32.4% for isolated vaginal cuff recurrence and 25% for other recurrence sites. The cancer-specific mortality-free survival rate was 32.4% for isolated vaginal cuff recurrence and 30.3% for the other recurrence sites [8].

#### Vaginal recurrence of leiomyosarcoma

Three cases of uterine leiomyosarcoma spread to the vagina have been reported in the literature. All had one lesion, and the complaint was vaginal bleeding in all. Therefore, patients underwent surgery [9].

#### Vaginal recurrence of breast cancer

Only 3 cases of breast cancer that had metastases in the vagina have been reported in the current literature. Only one had data available. The stage of this patient who underwent lumpectomy plus axillary lymphadenectomy for invasive lobular breast cancer was T2N1M0. Hormone therapy was administered for 5 years after adjuvant chemotherapy and RT. She presented with vaginal bleeding approximately three years after the treatment was completed. There was no metastasis in other regions except the vagina. The patient was treated with adjuvant liposomal doxorubicin after complete resection of the mass by modified vaginectomy [10].

#### Vaginal recurrence of pancreatic cancer

In the literature, there are three cases of pancreatic cancer with vaginal metastasis. In all three patients, the diagnosis of primary pancreatic adenocarcinoma was made at the time of evaluation due to symptoms arising from vaginal metastasis [11–13].

#### Vaginal recurrence of lung cancer

Only one case with vaginal metastasis of lung adenocarcinoma was reported in the literature. The patient admitted with frequent urination 2 years after surgery for lung cancer and metastasis in the vagina was detected [14].

#### Vaginal recurrence of salivary duct carcinoma

In the literature, there was only one case in which vaginal metastasis of salivary duct carcinoma was detected [15].

#### Vaginal recurrence of endometrial cancer

In a study, it was reported that vaginal recurrence occurred in 30 patients who underwent hysterectomy for EC, and the median time to recurrence after surgery was 20.6 months (range 2–219). The most common site of recurrence was the vaginal apex (60%) followed by the distal vagina (10%). All of the patients received salvage RT in combination with EBRT and vaginal brachytherapy in 24 patients and as a single modality in 6 patients. And, concurrent chemotherapy was applied in 20 patients during the RT period [16]. In another study, disease recurrence was documented in 14.5% (40/273) of patients with EC. The most frequent subtype was endometrioid subtype, and 30% of them had isolated vaginal relapses. The majority of patients had grade 2 and stage 1C disease at the time of initial diagnosis [17]. In a multicentre randomised study in which 714 patients with early-stage EC were evaluated, it was reported that isolated vaginal recurrence occurred in 5% of patients (37/714). While 30 of these patients did not receive RT at the time of initial diagnosis for EC, the remaining 7 received RT [18].

Carcinomas of the vagina are rare tumours involving approximately 1–3% of cancers occurring in the female genital tract [19]. The majority of vaginal malignancies are metastatic, often arising from the endometrium, cervix, vulva, ovary, breast, rectum, and kidney [20–23]. Vaginal metastases may occur by a direct extension (especially from endometrium, cervix, and vulva) or lymphatic or hematogenous spread [24, 25]. Ninety percent of EC are adenocarcinomas, and most primary vaginal cancers are SCC [26]. Therefore, histopathological examination is essential for an accurate diagnosis. Besides, when the literature was reviewed, we saw that the term of vaginal involvement could also be used for vaginal metastases, but we used the term vaginal metastasis because it is the most commonly used term for these patients.

## Conclusion

Standard therapy for vaginal metastasis has not yet been established. Therapeutic options for vaginal metastasis include surgical resection, RT, and chemotherapy, both in monotherapy and in combinations. While surgical resection and RT are frequently used in patients with isolated vaginal metastasis, systemic therapy is used in patients with extensive metastasis.

In conclusion, vaginal metastasis should be kept in mind in women with vaginal bleeding and a history of malignancy, and it is essential to perform a comprehensive gynaecological examination.

## Data Availability

Not applicable.

## Abbreviations

CRC: Colorectal cancers
CT: Computed tomography
EC: Endometrium carcinoma
EBRT: External beam radiotherapy
LVI: Lymphovascular invasion
MRI: Magnetic resonance imaging
RT: Radiotherapy
RCC: Renal cell cancer
SCC: Squamous cell carcinoma
TAH+BSO+BPPLND: Salpingo-oophorectomy and bilateral pelvic-paraaortic lymph node dissection
